# Epidemiological and virological surveillance of influenza viruses in China during 2020–2021

**DOI:** 10.1186/s40249-022-01002-x

**Published:** 2022-06-29

**Authors:** Wei-juan Huang, Yan-hui Cheng, Min-ju Tan, Jia Liu, Xi-yan Li, Xiao-xu Zeng, Jing Tang, He-jiang Wei, Tao Chen, Lei Yang, Yi-ran Xie, Jia-ying Yang, Ning Xiao, Da-yan Wang

**Affiliations:** National Institute for Viral Disease Control and Prevention, Chinese Center for Disease Control and Prevention; WHO Collaborating Centre for Reference and Research on Influenza; Key Laboratory for Medical Virology and Viral Diseases, National Health Commission, Beijing, China

**Keywords:** Influenza, B/Victoria lineage viruses, Epidemiological, Virological, Surveillance

## Abstract

**Background:**

During the coronavirus disease 2019 (COVID-19) pandemic, seasonal influenza activity declined globally and remained below previous seasonal levels, but intensified in China since 2021. Preventive measures to COVID-19 accompanied by different epidemic characteristics of influenza in different regions of the world. To better respond to influenza outbreaks under the COVID-19 pandemic, we analyzed the epidemiology, antigenic and genetic characteristics, and antiviral susceptibility of influenza viruses in the mainland of China during 2020–2021.

**Methods:**

Respiratory specimens from influenza like illness cases were collected by sentinel hospitals and sent to network laboratories in Chinese National Influenza Surveillance Network. Antigenic mutation analysis of influenza virus isolates was performed by hemagglutination inhibition assay. Next-generation sequencing was used for genetic analyses. We also conducted molecular characterization and phylogenetic analysis of circulating influenza viruses. Viruses were tested for resistance to antiviral medications using phenotypic and/or sequence-based methods.

**Results:**

In the mainland of China, influenza activity recovered in 2021 compared with that in 2020 and intensified during the traditional influenza winter season, but it did not exceed the peak in previous years. Almost all viruses isolated during the study period were of the B/Victoria lineage and were characterized by genetic diversity, with the subgroup 1A.3a.2 viruses currently predominated. 37.8% viruses tested were antigenically similar to reference viruses representing the components of the vaccine for the 2020–2021 and 2021–2022 Northern Hemisphere influenza seasons. In addition, China has a unique subgroup of 1A.3a.1 viruses. All viruses tested were sensitive to neuraminidase inhibitors and endonuclease inhibitors, except two B/Victoria lineage viruses identified to have reduced sensitivity to neuraminidase inhibitors.

**Conclusions:**

Influenza activity increased in the mainland of China in 2021, and caused flu season in the winter of 2021–2022. Although the diversity of influenza (sub)type decreases, B/Victoria lineage viruses show increased genetic and antigenic diversity. The world needs to be fully prepared for the co-epidemic of influenza and severe acute respiratory syndrome coronavirus 2 (SARS-CoV-2) virus globally.

**Supplementary Information:**

The online version contains supplementary material available at 10.1186/s40249-022-01002-x.

## Background

Influenza imposes a significant disease burden on the global population [1]. These annual epidemics are caused by the co-circulation of influenza A viruses and influenza B viruses in different proportions. Influenza A viruses cause the majority of influenza cases and are responsible for pandemics, but influenza B viruses is also an important cause of morbidity and mortality, and its prevention is one of the important global public health priorities [2, 3].

Unlike subtypes of influenza A viruses, which emerge periodically from animals, cause pandemics, and spread as single predominant antigenic variants, the Victoria and Yamagata lineages emerged as antigenic variants after the differentiation of influenza B viruses in the early 1980s, and both lineages have co-circulated globally since at least 2001 [4]. Since the first report of highly active influenza B viruses in Europe in 2015, a large number of epidemics have been reported worldwide, indicating significant changes in the evolution and epidemiology of influenza B viruses [5, 6]. Annual influenza vaccination is the main option for the prevention and control of influenza viruses [7, 8]. In 2012, the World Health Organization (WHO) began to recommend quadrivalent influenza vaccine, which contains two lineages of influenza B viruses; in contrast, trivalent vaccines, which contains only one of the lineages, often do not match the prevalent influenza B lineage in a given season, leading to low vaccine efficacy [9, 10].

Since the exponential global spread of severe acute respiratory syndrome coronavirus 2 (SARS-CoV-2) in March 2020, the number of influenza detections has declined dramatically [11, 12]. Global influenza activity has strengthened in 2021, but on the whole, remains below pre-pandemic levels, despite similar or even higher levels of influenza detection in many countries [13, 14]. Due to the travel restrictions caused by the coronavirus disease 2019 (COVID-19) and the impact of prevention and control measures in different countries or areas, it showed different influenza epidemic levels and epidemic dominant viruses, which are significantly different from those before the COVID-19 [13, 14]. In this study, we analyzed the epidemiological, antigenic and genetic characteristics and antiviral drug susceptibility of influenza viruses in the mainland of China from 2020 to 2021 years for better prevention and control of seasonal influenza in COVID-19.

## Methods

### Virus detection and isolation

Viruses isolated from specimens collected between week 1 in 2020 (January 1, 2020) and week 52 in 2021 (December 31, 2021) were obtained from the Chinese National Influenza Surveillance System.

The Chinese National Influenza Surveillance Network including 410 network laboratories and 554 sentinel hospitals, covers 32 provinces (including autonomous regions/municipalities) in the mainland of China. Sentinel hospitals collect respiratory specimens from influenza like illness (ILI) cases (body temperature ≥ 38 °C, accompanied by sore throat or cough), and sent them to network laboratories. Specimens tested positive by real time reverse transcription polymerase chain reaction (RT-PCR) were propagated in Madin–Darby canine kidney (MDCK) cells and/or embryonated chicken eggs by network laboratories. The viruses were submitted to Chinese National Influenza Center (CNIC) for further analysis. All the data mentioned above were submitted to the Chinese National Influenza Surveillance System by the members of the Chinese National Influenza Surveillance Network.

### Hemagglutination inhibition (HI) assays

Antigenic characteristics were based primarily on the results of HI tests, and HI titers less than or equal to four fold that of the homologous virus are considered antigenically similar. Specific test procedures were detailed in Manual for the Laboratory Diagnosis and Virological Surveillance of Influenza (WHO). The reference antisera used were the ferret antisera immunized with the reference viruses. These ferrets did not receive a booster immunization.

### Sequencing

Viral RNA was extracted by RNeasy Mini Kit (Qiagen, Dusseldorf, Germany). RT-PCR was performed using SuperScripthIII One-Step RT-PCR system (Thermo Fisher Scientific, Waltham, USA), and the reaction conditions and primers for RT-PCR and purification of products were performed as previously described [15]. The Qubit dsDNA HS assay system (Thermo Fisher Scientific, Waltham, USA) was used to determine the concentration of PCR products. Subsequently diluted to 1 ng/μl for library preparation using the Nextera XT DNA library preparation kits (Illumina, San Diego, USA). Next generation sequencing was performed using a MiSeq sequencing platform (Illumina, San Diego, USA), and sequences were obtained from FASTQ files using CLC Genomics Workbench software (Qiagen, Dusseldorf, Germany). Hemagglutinin (HA), neuraminidase (NA) and polymerase acidic (PA) sequences were submitted to the Global Initiative on Sharing All Influenza Data (GISAID).

### NA inhibition assay

Oseltamivir carboxylate (Hoffman-La Roche, Basel, Switzerland), zanamivir (GSK, Brentford, UK) were prepared in sterile distilled water and stored in aliquots at – 20 °C until use. NA inhibitors (NAIs) susceptibility was assessed using fluorescence-based NA enzyme inhibition assay, based the NA-Fluor™ kit (Applied Biosystems, Foster City, USA), to determine 50% inhibitory concentrations (IC_50_). The IC_50_ values were determined using GraphPad Prism software (version 5.00, GraphPad Software, La Jolla, USA). The interpretation of IC_50_ value was performed using the criteria of the World Health Organization Expert Working Group on Influenza Antiviral Susceptibility Monitoring (WHO-AVWG). The criteria for influenza B viruses: compared to the median IC_50_ value of all tested viruses by (sub)type and drug, normal inhibition (NI) with < 5-fold, reduced inhibition (RI) with 5- to 50-fold, and highly reduced inhibition (HRI) with > 50-fold [16].

### Phylogenic analysis

We downloaded global influenza B virus genomes (as of March 1, 2022) from GISAID databases, that with sample collection date from 2020 to 2021 years. Sequence alignment was constructed with Mafft software (version 7.490) [17], The Phyogenic tree was built in maximum-likehood method with rapid bootstrap replicates using FastTree software (version 2.1.11) [18].

## Results

### Epidemic of seasonal influenza in the mainland of China during 2020 and 2021

**Fig. 1:**
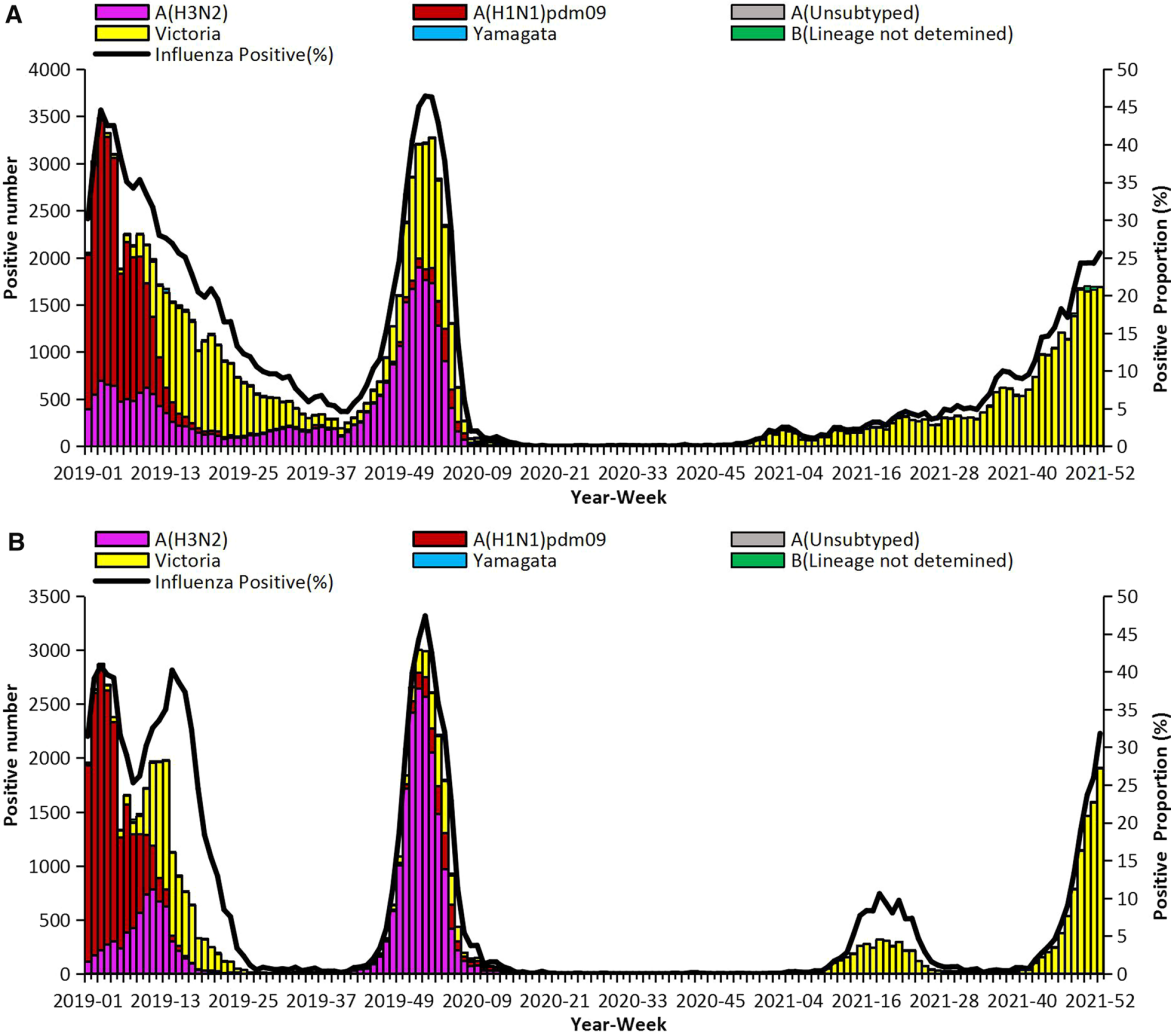
Influenza positive tests reported by network laboratories in the mainland of China during 2020 and 2021. **A** Southern China. **B** Northern China

The positive rate of specimens collected from ILI cases in northern China declined sharply from week 7 in 2020, and maintain a very low level in 2020, it increased slightly from week 10 of 2021 to week 26 of 2021, and began to increase consistently from week 42 of 2021, increasing to 31.8% by week 52 of 2021. Similarly in southern China, the positive rate decreased since week 7 in 2020 and increased slightly since week 49 in 2020, keep increasing to 25.7% by week 52 in 2021. In 2019–2020 influenza winter season, the week with the highest influenza positive rate was week 1, 2020, there is no obvious epidemic peak in the winter of 2020–2021 as in previous years, while the epidemic peak was “back” again in the winter of 2021–2022 (Fig. 1). Since September 2020, influenza B viruses accounted for the vast majority of influenza detections nationwide, and few influenza A viruses were detected in the mainland of China, despite the dominant of influenza A viruses in most other countries and areas in the same period.

In details, the network laboratories in southern China tested 294,656 specimens in 2020; among these specimens, 14,698 (5.0%) tested positive including 7881 (53.6%) for influenza A and 6817 (46.4%) for influenza B. Most of the positive samples (13,635, 92.8%) were collected in early 2020, from week 1 to week 6 of 2020, this period was the continuation of the 2019–2020 influenza winter season. Among the 7865 seasonal influenza A positive specimens that were subtyped, 1416 (18.0%) were influenza A(H1N1)pdm09 and 6449 (82.0%) were influenza A(H3N2). Among the 6719 influenza B viruses for which lineage was determined, 6704 (99.8%) belonged to the B/Victoria lineage and 15 (0.2%) belonged to the B/Yamagata lineage (Fig. 1A). In northern China, network laboratories tested 177,987 specimens for influenza in 2020, most (10,974, 93.5%) were collected during week 1 to 6 of 2020, with 11,742 (6.6%) were positive for an influenza virus: 9642 (82.1%) for influenza A and 2100 (17.9%) for influenza B. Among the 9636 seasonal influenza A viruses subtyped, 1542 (16.0%) were influenza A(H1N1)pdm09 and 8094 (84.0%) were influenza A(H3N2). Of the 2093 influenza B viruses with lineage determined, 2051 (98.0%) were B/Victoria and 42 (2.0%) were B/Yamagata (Fig. 1B).

In 2021, network laboratories in southern China tested 336,851 specimens for influenza; among these specimens, 25,227 (7.5%) tested positive including 64 (0.3%) for influenza A and 25,163 (99.7%) for influenza B. Among the 35 seasonal influenza A positive specimens that were subtyped, 14 (40.0%) were influenza A(H1N1)pdm09 and 21 (60.0%) were influenza A(H3N2). Among the 24,966 influenza B viruses for which lineage was determined, 24,953 (99.9%) belonged to the B/Victoria lineage and 13 (0.1%) belonged to the B/Yamagata lineage (Fig. 1A). Network laboratories in northern China tested 220,011 specimens in 2021. Among these, 12,117 (5.5%) were positive for influenza viruses, and influenza A and influenza B viruses were 11 (0.1%) and 12,106 (99.9%), respectively, of the tested viruses. Among the 6 seasonal influenza A viruses that were subtyped, 4 (66.7%) were influenza A(H1N1)pdm09 and 2 (33.3%) were influenza A(H3N2). Influenza B lineage information was available for 12,087 viruses; 12,074 (99.9%) were B/Victoria lineage and 13 (0.1%) were B/Yamagata lineage (Fig. 1B).

After January 2021, the COVID-19 pandemic, although a low number of influenza A(H1N1)pdm09, A(H3N2) and B/Yamagata viruses were detected by nucleic acid detection in the mainland of China, none have been confirmed by sequencing or virus isolation.

### Genetic characterization of influenza B viruses

Among the 1137 B/Victoria lineage viruses with HA gene segments sequenced and phylogenetically analyzed, all belonged to genetic clade 1A, and nearly all (99.2%, 1128/1137) were in subclade 1A.3, which has a triple amino acid deletion (positions 162–164) and the substitution K136E in HA, and in which, 92.6% (1053/1137) belonged to group 1A.3a characterized by HA amino acid substitutions N150K, G184E, N197D, and R279K, and was further divided into subgroup 1A.3a.1 (614, 58.3%), which had additional HA amino acid substitutions V220M and P241Q, and subgroup 1A.3a.2 (439, 41.7%), which had HA amino acid substitutions A127T, P144L, and K203R.

**Fig. 2:**
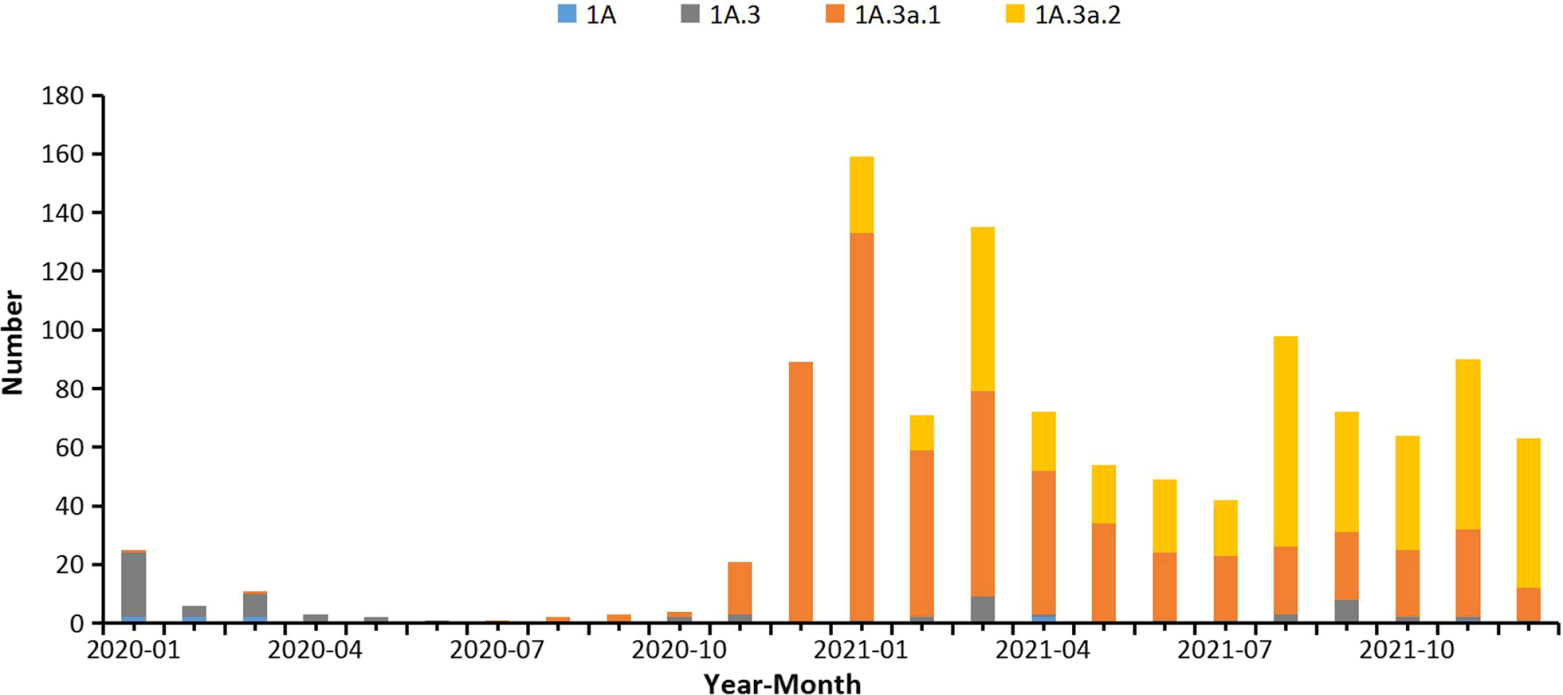
Distribution of HA gene clades of B/Victoria lineage viruses isolated in the mainland of China over time during 2020 and 2021

**Fig. 3:**
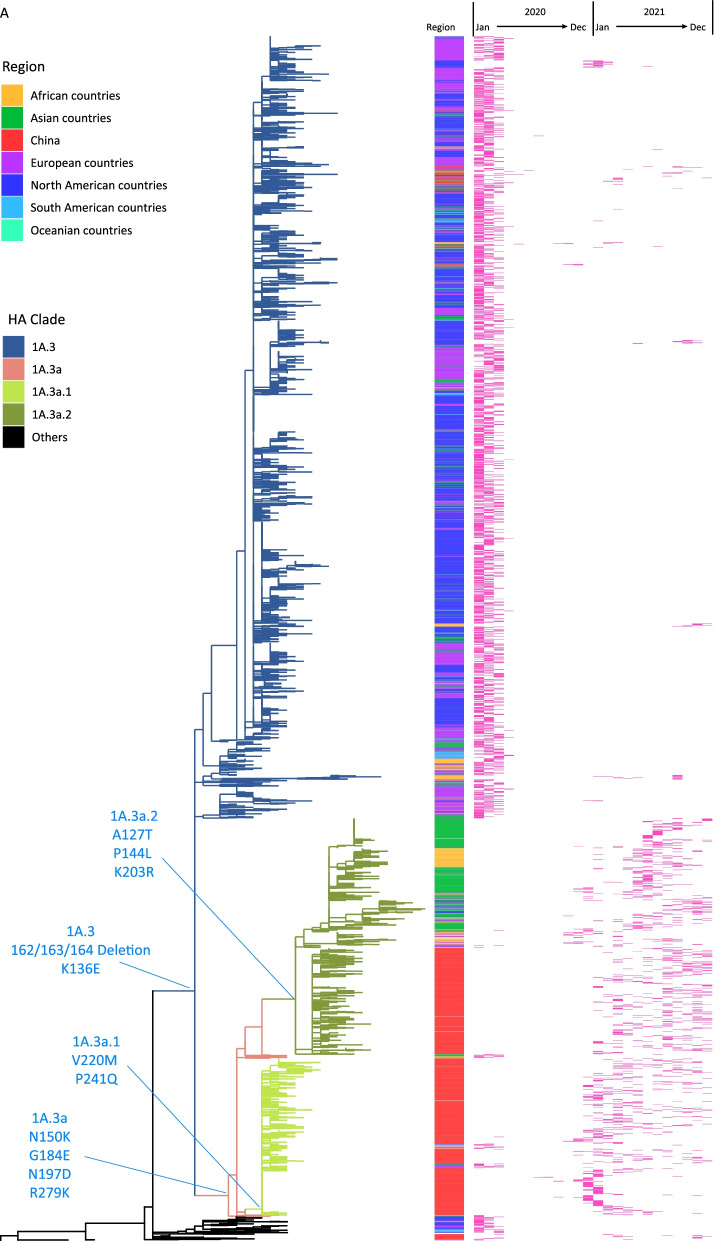

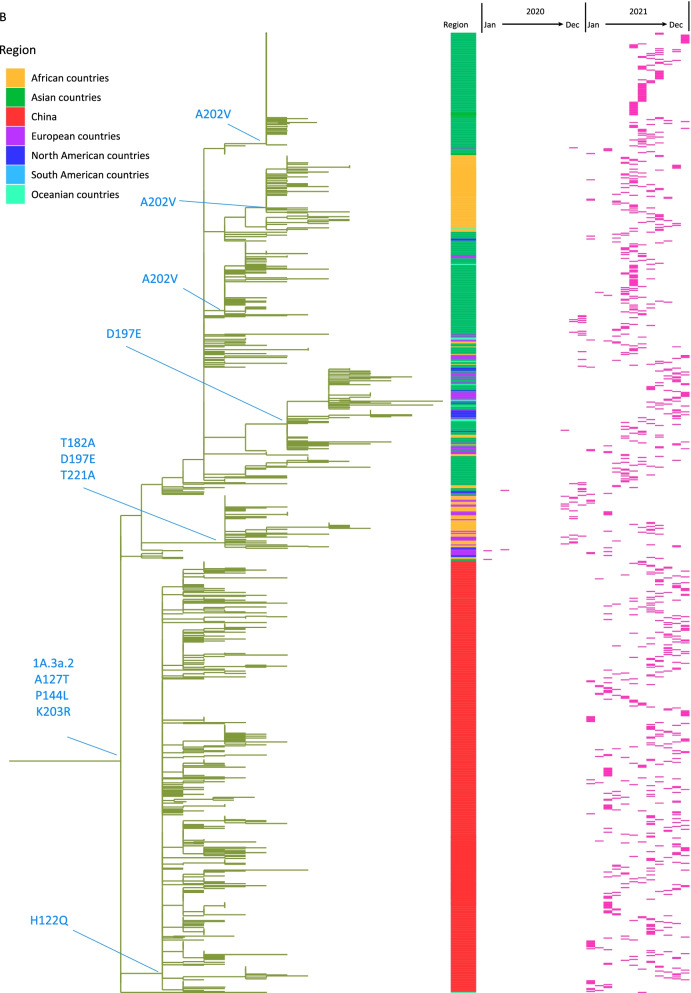
Phylogenic tree of the HA gene of the global B/Victoria lineage viruses from 2020 to 2021. **A** HA phylogenic tree of all sequence. **B** HA phylogenic tree of Subgroup 1A.3a.2. HA sequences after removing duplicates and incomplete coding regions, a total of 5046 sequences were used, including 1137 sequences in the mainland of China. The red bar denotes the distribution of viruses collection months. Asia countries excludes the mainland of China

By searching the sequences in GISAID, subgroup 1A.3a.1 viruses were almost exclusively detected in the mainland of China and predominated from November 2020. Subgroup 1A.3a.2 viruses were first detected in Jiangxi Province in January 2021 and accounted for a higher proportion than 1A.3a.1 from August 2021 and onwards (Fig. 2). Subgroup 1A.3a.2 viruses have circulated in Asia, Africa, Europe, Oceania and North America, but shown further genetic divergence, with additional HA amino acid substitutions encoded in viruses from different geographic locations, which is also a special feature after COVID-19. 1A.3a.2 viruses from the mainland of China encode a specific additional HA amino acid substitution H122Q, viruses from South Asia and Africa have A202V, and more recently viruses from Africa, Asia, Europe and North America have D197E, while a subset of previously circulating viruses from Africa, Europe, West Asia and North America encode the additional amino acid substitutions T182A, D197E and T221A (Fig. 3).

**Fig. 4:**
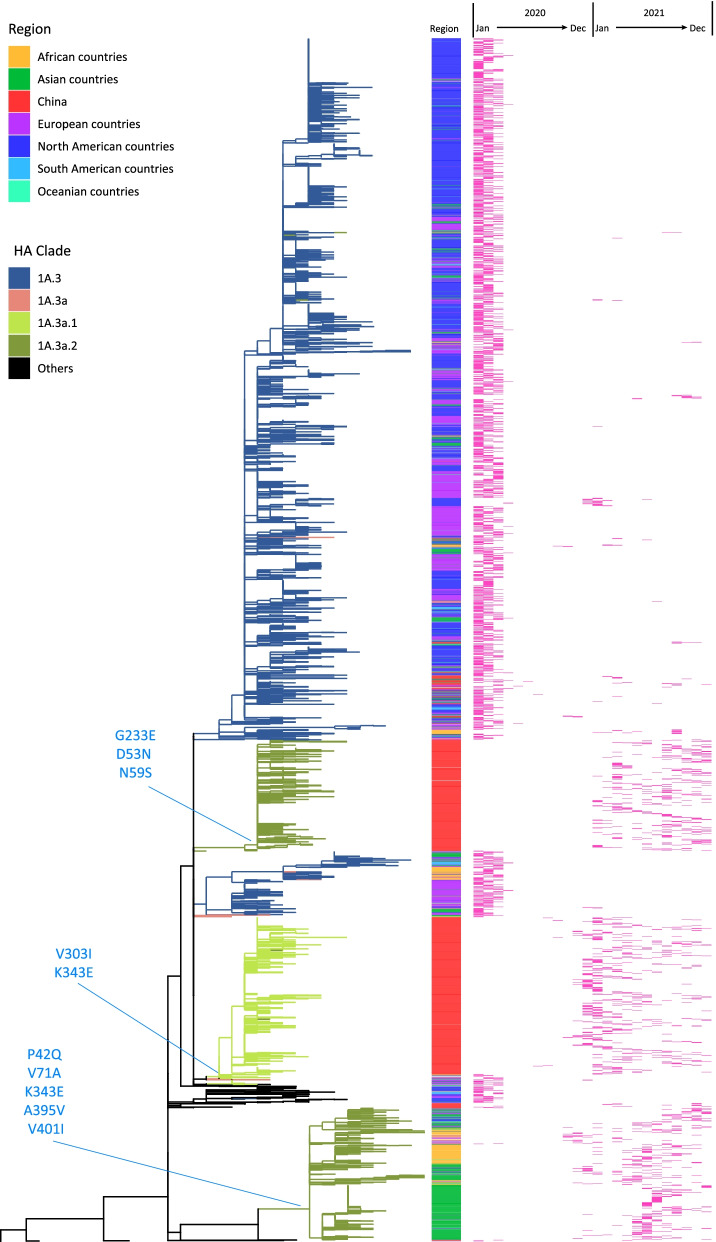
Phylogenic tree of the NA gene of the global B/Victoria lineage viruses from 2020 to 2021. NA sequences after removing duplicates and incomplete coding regions, a total of 4743 sequences were used, including 1137 sequences in the mainland of China. The red bar denotes the distribution of viruses collection months. Asia countries excludes the mainland of China

Phylogenetic analysis of B/Victoria lineage NA genes demonstrated that most NA genes cluster with viruses expressing the same HA clade. 1A.3a.1 viruses collected in the mainland of China possess NA with substitutions V303I and K343E. 1A.3a.2 viruses possess NA genes which cluster in at least three separate clades. The majority of 1A.3a.2 viruses from Chinese cluster possess NA substitutions D53N, N59S and G233E. In other global regions, a few 1A.3a.2 viruses cluster with viruses expressing HA of 1A.3, but many 1A.3a.2 viruses cluster with viruses expressing HA of 1A with encoding NA additional substitutions P42Q, V71A, K343E, A395V and V401I, suggesting the possibility of reassortment (Fig. 4).

More insertion or deletion mutations have been found in influenza B viruses during 2020–2021 than in previous years. Among the B/Victoria lineage viruses sequenced in the mainland of China in 2021, 14 viruses had 167N amino acid inserted in the HA, which belonged to 1A.3a.2, and all but one virus were isolated from Gansu Province; one virus had 185E amino acid deleted in the HA, which belonged to 1A.3a.1. Ten viruses had 73L amino acid inserted in the NA, distributed in 1A.3, 1A.3a.1, and also detected in 1A.3a.2, which predominated in other global regions, especially in Asia.

### Antigenic characterization of influenza B viruses

Antigenic characterization of the 4182 B/Victoria lineage viruses were conducted using HI tests. A total of 1582 (37.8%) B/Victoria lineage viruses were recognized well by the ferret antisera raised against B/Washington/02/2019 (subclade 1A.3), the egg-propagated reference virus representing the B/Victoria lineage component of the 2020–2021 and 2021–2022 Northern Hemisphere influenza vaccines [19, 20]. 45.6% viruses (1798/3939) were recognized well by ferret antisera raised egg-propagated against B/Sichuan-Jingyang/12048/2019 (subgroup 1A.3a.1). Of the 1673 viruses isolated since August 2021, 1474 viruses (88.1%) were recognized well by ferret antisera raised against B/Austria/1359417/2021 (subgroup 1A.3a.2), the egg-propagated reference virus representing the B/Victoria lineage component of the 2022 Southern Hemisphere and 2022–2023 Northern Hemisphere influenza vaccines [13, 14] (Table 1).

**Table 1:** Summary of antigenic analysis of B/Victoria lineage viruses isolated in the mainland of China by month during 2020 and 2021

Collection time	Reference Ferret Antisera								
	B/Washington/02/2019 (1A.3)			B/Sichuan-Jingyang/12048/2019 (1A.3a.1)			B/Austria/1359417/2021 (1A.3a.2)		
	No. of like^a^(%)	No. of low^b^ (%)	Total	No. of like (%)	No. of low (%)	Total	No. of like (%)	No. of low (%)	Total
2020.1	181 (96.3)	7 (3.7)	188	0 (0)	1 (100)	1	–	–	–
2020.2	29 (87.9)	4 (12.1)	33	0 (0)	3 (100)	3	–	–	–
2020.3	22 (84.6)	4 (15.4)	26	0 (0)	3 (100)	3	–	–	–
2020.4	4 (100)	0 (0)	4	0 (0)	1 (100)	1	–	–	–
2020.5	4 (100)	0 (0)	4	1 (25.0)	3 (75.0)	4	–	–	–
2020.6	4 (100)	0 (0)	4	0 (0)	4 (100)	4	–	–	–
2020.7	0 (0)	1 (100)	1	1 (100)	0 (0)	1	–	–	–
2020.8	0 (0)	2 (100)	2	2 (100)	0 (0)	2	–	–	–
2020.9	0 (0)	3 (100)	3	3 (100)	0 (0)	3	–	–	–
2020.10	4 (80.0)	1 (20.0)	5	2 (40.0)	3 (60.0)	5	–	–	–
2020.11	17 (73.9)	6 (26.1)	23	18 (78.3)	5 (21.7)	23	–	–	–
2020.12	30 (30.9)	67 (69.1)	97	97 (100)	0 (0)	97	–	–	–
2021.1	79 (36.1)	140 (63.9)	219	212 (96.8)	7 (3.2)	219	–	–	–
2021.2	105 (68.2)	49 (31.8)	154	138 (89.6)	16 (10.4)	154	–	–	–
2021.3	153 (48.4)	163 (51.6)	316	245 (77.5)	71 (22.5)	316	–	–	–
2021.4	88 (28.0)	226 (72.0)	314	267 (85.0)	47 (15.0)	314	–	–	–
2021.5	90 (30.0)	210 (70.0)	300	231 (77.0)	69 (23.0)	300	–	–	–
2021.6	73 (29.3)	176 (70.7)	249	173 (69.5)	76 (30.5)	249	–	–	–
2021.7	48 (24.7)	146 (75.3)	194	93 (47.9)	101 (52.1)	194	–	–	–
2021.8	76 (35.7)	137 (64.3)	213	55 (25.8)	158 (74.2)	213	2 (100)	0 (0)	2
2021.9	147 (48.8)	154 (51.2)	301	132 (43.9)	169 (56.1)	301	117 (84.2)	22 (15.8)	139
2021.10	110 (32.4)	230 (67.6)	340	147 (43.2)	193 (56.8)	340	307 (90.3)	33 (9.7)	340
2021.11	155 (23.2)	512 (76.8)	667	172 (25.8)	495 (74.2)	667	581 (87.1)	86 (12.9)	667
2021.12	161 (30.7)	364 (69.3)	525	152 (29.0)	373 (71.0)	525	467 (89.0)	58 (11.0)	525
Total	1582 (37.8)	2602 (62.2)	4182	2141 (54.4)	1798 (45.6)	3939	1474 (88.1)	199 (11.9)	1673

“–”: Tests not performed

^a^Less than or equal to fourfold compared to homologous HI titers

^b^More than or equal to eightfold compared to homologous HI titers

Viruses from 1A.3, 1A.3a.1 and 1A.3a.2 were antigenically different. Antigenic analysis showed that subclade 1A.3, mostly isolated from the early 2020 years, were recognized well by ferret antisera raised against B/Washington/02/2019 (1A.3), while the much greater numbers of viruses from subgroups 1A.3a.1 and 1A.3a.2 were recognized poorly. Post-infection ferret antisera raised against B/Sichuan-Jingyang/12048/2019 (1A.3a.1) recognized viruses in subgroup 1A.3a.1 well but recognized subgroup 1A.3a.2 viruses less well. Post-infection ferret antisera raised against B/Austria/1359417/2021 (1A.3a.2) recognized viruses from same subgroup well but recognized other viruses less well.

The viruses with insertion or deletion of amino acid in the HA and NA detected in 2021 years, had limited impact on antigenicity (Table 2).

**Table 2:** Antigenic analysis of viruses with deletions or insertions in HA or NA genes of B/Victoria lineage viruses detected in the mainland of China in 2021

		Reference Ferret Antisera														
		1A	1A.1	1A.3		1A.3a.1		1A.3a.2								
		MDCK	MDCK	EGG	MDCK	EGG	MDCK	EGG	MDCK	EGG	MDCK	EGG	MDCK			
Reference virus		BS/60	CO/6	WT/2	WT/2	SCH/12048	SCH/12048	HEN/1118	HEN/1118	GS/1281	GS/1281	AUS/1359417	MI/1	HA clade	Collection date	Passage history
1	B/Brisbane/60/2008	**320**	80	80	40	< 10	< 10	< 10	20	< 10	< 10	10	< 10	1A	2008/8/4	M4/C2 + 2
2	B/Colorado/06/2017	80	**80**	40	20	< 10	< 10	< 10	10	< 10	< 10	10	< 10	1A.1	2017/6/13	C2
3	B/Washington/02/2019	80	80	**160**	160	40	20	10	20	< 10	< 10	20	40	1A.3	2019/1/4	E4
4	B/Washington/02/2019	80	40	80	**160**	20	< 10	10	< 10	< 10	< 10	20	40	1A.3	2019/1/4	C4
5	B/Sichuan-Jingyang/12048/2019	40	20	80	40	**640**	640	160	160	640	40	80	160	1A.3a.1	2019/11/26	E2
6	B/Sichuan-Jingyang/12048/2019	40	10	80	20	640	**320**	80	80	160	20	80	80	1A.3a.1	2019/11/26	C3
7	B/Henan-Xigong/1118/2021	< 10	10	20	20	80	80	**160**	320	640	160	160	320	1A.3a.2	2021/2/17	E2
8	B/Henan-Xigong/1118/2021	20	20	40	20	80	160	320	**640**	1280	320	320	640	1A.3a.2	2021/2/17	C2
9	B/Gansu-Baiyin/1281/2021	10	20	40	< 10	160	160	640	640	**1280**	640	640	640	1A.3a.2 (insert HA-167N)	2021/4/13	E2
10	B/Gansu-Baiyin/1281/2021	10	< 10	40	< 10	80	160	640	640	1280	**640**	640	640	1A.3a.2 (insert HA-167N)	2021/4/13	C2
11	B/Austria/1359417/2021	10	40	80	40	160	160	640	640	1280	640	**640**	1280	1A.3a.2	2021/1/9	E4
12	B/Michigan/01/2021	20	20	40	40	80	160	640	640	1280	320	640	**1280**	1A.3a.2	2021/1/9	C3

Test virus																
13	B/Gansu-Minxian/330/2021	20	< 10	20	< 10	80	80	320	640	1280	160	320	640	1A.3a.2 (insert HA-167N)	2021/3/15	C3
14	B/Gansu-Chengguan/1515/2021	20	10	40	< 10	80	160	640	640	1280	640	640	640	1A.3a.2 (insert HA-167N)	2021/3/30	C2
15	B/Gansu-Chengguan/1490/2021	20	10	40	< 10	40	160	320	640	1280	320	640	640	1A.3a.2 (insert HA-167N)	2021/3/24	C2
16	B/Gansu-Chengguan/1474/2021	20	< 10	20	< 10	40	80	320	160	1280	320	320	320	1A.3a.2 (insert HA-167N)	2021/3/22	C2
17	B/Gansu-Anding/1194/2021	20	< 10	20	< 10	40	40	320	320	1280	320	320	640	1A.3a.2 (insert HA-167N)	2021/3/10	C3
18	B/Gansu-Baiyin/1269/2021	10	20	40	< 10	160	160	640	640	320	640	320	640	1A.3a.2 (insert HA-167N)	2021/4/6	E1
19	B/Gansu-Baiyin/1252/2021	10	< 10	20	< 10	40	80	320	160	1280	320	160	320	1A.3a.2 (insert HA-167N)	2021/3/31	C2
20	B/Gansu-Baiyin/1224/2021	10	< 10	20	< 10	40	80	160	320	180	160	320	640	1A.3a.2 (insert HA-167N)	2021/3/23	C2
21	B/Gansu-Baiyin/1209/2021	10	< 10	10	< 10	40	80	320	160	1280	320	320	320	1A.3a.2 (insert HA-167N)	2021/3/16	C2
22	B/Qinghai-Chengdong/1404/2021	10	< 10	20	< 10	80	80	320	320	1280	320	320	320	1A.3a.2 (insert HA-167N)	2021/4/7	C2
23	**B/Gansu-Baiyin/1293/2021**													1A.3a.2 (insert HA-167N)	2021/4/14	Not done
24	**B/Gansu-Chengguan/1516/2021**													1A.3a.2 (insert HA-167N)	2021/3/30	No virus^a^
25	**B/Gansu-Chengguan/1480/2021**													1A.3a.2 (insert HA-167N)	2021/3/22	No virus^a^
26	B/Jiangxi-Zhushan/1164/2021	40	20	80	20	640	320	80	80	320	20	80	80	1A.3a.1 (delete HA-185E)	2021/3/1	C3
27	B/Shanghai-Songjiang/1193/2021	40	20	80	40	640	320	80	80	320	40	80	160	1A.3a.1 (insert NA-73L)	2021/3/11	C2
28	B/Hubei-Chongyang/215/2021	< 10	< 10	20	20	640	320	80	80	160	20	80	80	1A.3a.1 (insert NA-73L)	2021/4/26	E2
29	B/Hubei-Chongyang/286/2021	40	10	80	20	640	320	40	80	160	20	80	80	1A.3a.1 (insert NA-73L)	2021/5/24	C2
30	B/Sichuan-Qingyang/11987/2021	10	< 10	40	< 10	80	80	320	160	1280	160	640	640	1A.3a.2 (insert NA-73L)	2021/10/12	C2
31	B/Jiangsu-Quanshan/11755/2021	< 10	< 10	20	10	80	80	320	320	1280	320	160	320	1A.3a.2 (insert NA-73L)	2021/11/3	E3
32	B/Gansu-Wudou/1305/2021	80	40	80	320	20	< 10	< 10	20	< 10	< 10	20	40	1A.3 (insert NA-73L)	2021/3/12	C3
33	**B/Gansu-Wudou/1280/2021**													1A.3 (insert NA-73L)	2021/3/11	No virus^a^
34	**B/Gansu-Wudou/1348/2021**													1A.3 (insert NA-73L)	2021/3/22	No virus^a^
35	**B/Gansu-Wudou/1312/2021**													1A.3 (insert NA-73L)	2021/3/15	No virus^a^
36	**B/Gansu-Wudou/1361/2021**													1A.3 (insert NA-73L)	2021/3/28	Not done

Homologous titers are underlined and bolded

Viruses without antigenic analysis results are marked in bold

E: Egg isolates; C: MDCK cell isolates

^a^No virus was isolated from clinical specimens

### Antiviral susceptibility of influenza B viruses

During 2020 and 2021 years, a total of 369 and 1622 B/Victoria lineage viruses, respectively were assessed for NAI susceptibility by CNIC using phenotypic and/or NA sequence-based methods. Of these respective total numbers, 367 viruses in 2020 and 1140 in 2021 were assessed for susceptibility to oseltamivir and zanamivir by NA inhibition assays. The potential NAI susceptibility of remaining viruses, 2 in 2020 and 482 in 2021, were assessed only based on NA sequence analysis.

One virus (B/Guangxi-Qinbei/32/2021) displayed RI by zanamivir, which was conferred by the substitution of H273Q in NA, this substitution was not reported previously. One virus (B/Henan-Xigong/1515/2021) were found to contain D197N substitution in NA based on sequence analysis. Thus, all 369 viruses in 2020 were sensitive to NAIs, while 0.1% (2/1622) viruses in 2021 were identified as having RI by at least one NAI.

A total of 1134 B/Victoria lineage viruses were screened for susceptibility to the endonuclease inhibitor, baloxavir, by sequence-based analysis, and none showed evidence of reduced susceptibility.

## Discussion

In response to the COVID-19 pandemic, influenza surveillance and/or reporting activities have had varying degrees of impact in many countries. SARS-CoV-2 mitigation strategies including travel restrictions, use of respiratory protection, and social isolation measures in most countries have led to a reduction in influenza transmission. The circulation of influenza viruses showed historically low levels in 2020 [19, 20]. In 2021, SARS-CoV-2 continued to circulate globally, however, influenza epidemics were reported by a number of countries and regions, with higher detections of influenza activity in 2021–2022 winter season than in 2020–2021 season, particularly in Europe, North America, Africa and China [14]. Globally, influenza A viruses were detected in more countries in 2021, while influenza B viruses were reported to be dominant in China and few other countries [14].

The influenza epidemic levels in the southern and northern provinces are often different in the same period, most importantly due to different climatic and geographical factors [21], so we analyzed the influenza activities in the southern and northern provinces respectively.

Compared to previous seasons, the diversity of (sub)type of influenza virus co-circulating has decreased in China since COVID-19, with nearly all of them are B/Victoria lineage viruses [22, 23]. However, significant increased genetic diversity was seen in circulating B/Victoria lineage virus during this period. After 1A.3a.1 viruses predominated in late 2020, the proportion detected has continued to increase since 1A.3a.2 viruses were first detected in the southern China in January 2021, with a higher proportion than 1A.3a.1 viruses since August 2021, while 1A and 1A.3 viruses have been consistently prevalent at low proportions. 1A.3a.1 viruses were almost exclusively detected in China. It is not clear whether the acceleration of genotype differentiation of B/Victoria lineage viruses is related to the reduction of subtypes of influenza virus.

The observed HA deletion variants were not unprecedented in influenza B viruses [24]. The ancestral B/Yamagata lineage viruses, which was prevalent before 1988 carried double (^160^VPK*D*N^166^) or triple (^160^VP***KN^166^) deletions. In contrast, no deletion was observed in the ancestral Victoria viruses [24]. Therefore, it is not surprising that the HA deletions (^160^VP**DKN^166^ or ^160^VP***KN^166^) were present in recent B/Victoria lineage viruses. The deletion of influenza B viruses in the antigenic loop 160 of the HA protein may be critical for their antigenicity [25]. However, all of those detected in the mainland of China in 2021 had a 167N amino acid insertion in the HA gene and only one virus had a 185E deletion, but the antigenicity of these viruses did not differ significantly from the same clade by antigenic analysis.

1A.3, 1A.3a.1 and 1A.3a.2 viruses were antigenically distinct, 62.2% tested viruses were recognized poorly by the ferret antisera raised against B/Washington/02/2019 (1A.3), the egg-propagated reference virus representing the B/Victoria lineage component of the 2020–2021 and 2021–2022 Northern Hemisphere influenza vaccines [19, 20]. It is worth noting that the effectiveness of vaccines cannot be based solely on the similarity of the circulating viruses with the reference vaccine viruses, there are many other factors.

Based on our analysis, nearly all tested viruses for susceptibility to therapeutics were susceptible to neuraminidase and endonuclease inhibitors. Only two viruses assessed in 2021 were identified as having reduced susceptibility by at least one NAI using phenotypic and/or NA sequence-based methods. One virus had NA-D197N substitution and this substitution was reported previously as a borderline NI/RI phenotype [26]. One virus showed RI by zanamivir, with the NA-H273Q substitution, which has not been described before. However, the NA-H273Y substitution, which was previously reported in influenza B viruses, associated with NI/RI to oseltamivir [27]. We should take further assessment and evaluation to clarify the significance of the substitution at 273 position. Therefore, NAI and baloxavir remain appropriate options for the treatment of influenza virus infections, but close monitoring of antiviral drug susceptibility is required.

The surveillance data presented here remind us that influenza activity is recovering in 2021, although not yet reached the highest level before COVID-19 pandemic. Especially after the significant restart of international travel, the chronic lack of sustained natural exposure to other subtypes of influenza viruses may affect the severity of the next influenza season. Maintaining influenza surveillance and outbreak response is therefore critical to track the geographic spread of the virus and its variant characteristics, and influenza surveillance should continue to be strengthened to maintain vaccine optimization for circulating influenza viruses.

The viruses we used for analysis were sent by the members of the Chinese National Influenza Surveillance Network. The isolation of the viruses may be delayed and suspended due to the impact of the COVID-19 pandemic, potentially leading to biased results in our study.

## Conclusions

Influenza activity in the mainland of China declined sharply at the beginning of the COVID-19 pandemic and gradually increased in 2021, although activity intense remained lower than prior to the pandemic. The diversity of influenza virus (sub)type circulating has decreased since COVID-19 compared to previous seasons, with predominated almost exclusively B/Victoria lineage viruses, while the genetic diversity of B/Victoria lineage viruses has increased significantly during this period. Antigenic analysis with ferret antiserum showing that only 37.8% of tested viruses were antigenically similar to reference viruses representing the components of the vaccine for the 2020–2021 and 2021–2022 Northern Hemisphere influenza seasons. Influenza surveillance should be continuously strengthened, and there is an urgent need to make full preparations for the co-epidemic of influenza and SARS-CoV-2 virus globally.

## Supplementary Information

**Additional file 1.** The list of southern and northern provinces in the mainland of China

## Data Availability

The data presented in this study are available on reasonable request from the corresponding author.

## Abbreviations

COVID-19: Coronavirus disease 2019
WHO: World Health Organization
SARS-CoV-2: Severe acute respiratory syndrome coronavirus 2
ILI: Influenza like illness
RT-PCR: Reverse transcription polymerase chain reaction
MDCK: Madin–Darby canine kidney
CNIC: Chinese National Influenza Center
HI: Hemagglutination inhibition
HA: Hemagglutinin
NA: Neuraminidase
PA: Polymerase acidic
GISAID: Global Initiative on Sharing All Influenza Data
NAI: NA inhibitor
IC_50_: 50% Inhibitory concentrations
WHO-AVWG: World Health Organization Expert Working Group on Influenza Antiviral Susceptibility Monitoring
NI: Normal inhibition
RI: Reduced inhibition
HRI: Highly reduced inhibition
