# Supplementary material for: Epidemiological and virological surveillance of influenza viruses in China during 2020–2021
Source: Infect Dis Poverty. 2022 Jun 29;11:74. doi: 10.1186/s40249-022-01002-x (PMC9244124; doi:10.1186/s40249-022-01002-x)
Supplement: Supplementary file 1 — Additional file 1. The list of southern and northern provinces in the mainland of China [file 40249_2022_1002_MOESM1_ESM.docx]

| **Northern provinces** | **Southern provinces** |
| --- | --- |
| Henan | Zhejiang |
| Shandong | Guangdong |
| Heilongjiang | Hunan |
| Beijing | Yunnan |
| Hebei | Guangxi |
| Tianjin | Chongqing |
| Liaoning | Jiangsu |
| Shaanxi | Anhui |
| Inner Mongolia | Guizhou |
| Qinghai | Sichuan |
| Xinjiang | Fujian |
| Xinjiang Construction Corps | Hubei |
| Gansu | Jiangxi |
| Shanxi | Hainan |
| Ningxia | Shanghai |
| Jilin |  |
| Tibet |  |

**Additional** **file 1** The list of southern and northern provinces in the mainland of China
